# Development of Fabrication Methods of Filler/Polymer Nanocomposites: With Focus on Simple Melt-Compounding-Based Approach without Surface Modification of Nanofillers

**DOI:** 10.3390/ma3031593

**Published:** 2010-03-04

**Authors:** Mitsuru Tanahashi

**Affiliations:** Department of Molecular Design and Engineering, Graduate School of Engineering, Nagoya University, Furo-cho, Chikusa-ku, Nagoya 464-8603, Japan; E-Mail: mtana@numse.nagoya-u.ac.jp; Tel.: +81-52-789-3361; Fax: +81-52-789-3228

**Keywords:** nanocomposite, thermoplastic polymer, silica nanoparticle, agglomerate, pore structure, packing arrangement, fracture strength, colloidal stability, shear stress, direct melt-compounding

## Abstract

Many attempts have been made to fabricate various types of inorganic nanoparticle-filled polymers (filler/polymer nanocomposites) by a mechanical or chemical approach. However, these approaches require modification of the nanofiller surfaces and/or complicated polymerization reactions, making them unsuitable for industrial-scale production of the nanocomposites. The author and coworkers have proposed a simple melt-compounding method for the fabrication of silica/polymer nanocomposites, wherein silica nanoparticles without surface modification were dispersed through the breakdown of loose agglomerates of colloidal nano-silica spheres in a kneaded polymer melt. This review aims to discuss experimental techniques of the proposed method and its advantages over other developed methods.

## 1. Introduction

Polymer-matrix composites containing inorganic fillers (filler/polymer composites) have been receiving significant attention lately because of their interesting and useful characteristics, such as good mechanical properties, thermal resistance and chemical reagent resistance [1]. Due to recent developments in the field of nanotechnology, there has been growing interest in polymer-matrix composites in which nano-sized fillers are distributed homogeneously (known as filler/polymer nanocomposites), due to their unique optical, electric and magnetic properties, as well as their dimensional and thermal stability [2,3,4,5,6,7,8,9,10,11]. The nanocomposite materials are also potential candidates for catalysts [12,13], gas-separation membranes [14,15,16], contact lenses [17] and bioactive implant materials [18,19]. The traditional practical method for dispersing inorganic nanofillers in polymer matrices is direct melt-compounding of the polymer with the fillers [18,19,20,21,22,23,24,25]. However, the surface activity of the nanofillers is extremely high, and the particles consequently have a tendency to aggregate tightly, creating micron-sized filler-clusters. This is one of the major problems in the fabrication of filler/polymer nanocomposites. In view of this issue, there have been a number of attempts to disperse nanofillers uniformly in polymer matrices by using methods with organic modification of the surface or interlayer of nanofillers and a variety of sol-gel and/or polymerization reactions [2,3,4,5,6,7,8,9,10,11,12,13,14,15,16,17,19,23,26,27,28,29,30,31,32,33,34,35,36,37,38,39,40,41,42,43,44,45,46,47,48,49,50,51,52,53,54,55,56,57,58,59]. However, these technologies require complicated chemical reactions making them unsuitable for industrial-scale production of nanocomposites with a wide volume fraction range of nanofillers and various combinations of filler and polymer material species. From the viewpoint of the fabrication of high performance particle/polymer nanocomposites on an industrial scale, the author and coworkers have developed a simple method for dispersing inorganic nanoparticles into various polymers by direct melt-compounding, without requiring any surface modification of the nanoparticles or complicated reactions [60,61,62,63,64,65,66].

This paper reviews the conventional and developed methods for the fabrication of filler/polymer nanocomposites, particularly the experimental techniques of the simple melt-compounding method proposed by the author’s research group. On the basis of the main findings of the authors’ previous studies on the dispersion of nano-sized spherical silica particles in some thermoplastic polymers, the advantages of this method over other developed methods are also described.

## 2. General Methods for the Fabrication of Filler/Polymer Nanocomposites

The general methods applied to the fabrication of nanocomposites can be classified into the following four approaches. The first approach is an intercalation method based on the exfoliation of layered silicates such as montmorillonite and mica. The second is *in situ* polymerization in the presence of the nanofillers, and the third is both *in situ* formation of the nanofillers and *in situ* polymerization. The fourth is a direct mechanical mixing of the polymer and nanofillers. In this chapter, the features of some typical methods developed by both academic and industrial researchers are summarized.

### 2.1. Intercalation Method

The intercalation method is a typical top-down approach based on the downsizing of fillers to nanodimensions. In this method, the exfoliation of the layered silicates used as the inorganic filler occurs by intercalating an organic compound into the interlayer space of the silicate, resulting in the uniform dispersion of plate-like nanofillers [2,37,38,39,40,41,42,43,44,45,46,47,48,49,50]. The layered silicate must be organically modified by organic surfactants containing quaternary cation functionality, such as amino acids, alkylammonium, imidazolium and phosphonium salts, to achieve enough hydrophobicity to be miscible with the organic compounds because the silicate is hydrophilic, but the organic compound is hydrophobic [37,38,39,40]. The intercalation of polymeric materials into the organically modified layered silicates and the subsequent exfoliation of the silicates are generally performed utilizing a chemical or mechanical technique. The chemical technique is *in situ* polymerization of the monomers within the silicate layers (*in situ* intercalative polymerization method) [2,40,41,42,43]. Shioyama *et al.* have found that *in situ* polymerization of the monomers could also occur in the interlayer spacing of graphite with a similar layered structure to that of the above-mentioned silicate [44,45]. These results raise the possibility of the fabrication of graphite/polymer nanocomposites by the *in situ* intercalative polymerization method. On the other hand, the mechanical technique is a direct intercalation of the polymer with layered silicates in a suitable solvent [42,43,46,47] or a melt intercalation method *via* melt-compounding of the polymer with the silicate in the absence of a solvent under a high shear condition [42,43,48,49,50]. In this method, even polymeric systems unsuitable for *in situ* polymerization can be used for the fabrication of nanocomposites.

### 2.2. In Situ Polymerization Method

This method, as well as *in situ* intercalative polymerization, employs polymerization reactions. In the method, inorganic nanoparticles are dispersed in the monomer or monomer solution, and the resulting mixture is polymerized by standard polymerization methods [2,11,29,30,31,32,33,34,51]. Metal/polymer nanocomposites have been synthesized *via* the simultaneous formation of metal particles from their suitable metal precursors and matrix polymers [52,53]. The reactions occur in the presence of a protective polymer, which limits the size of the metal particles. In the case where organometallic complexes, such as palladium, platinum, silver and gold, are dissolved in the monomer, precious metal-cluster/polymer nanocomposites can be synthesized *via* the polymerization and subsequent reduction of the metal ions in the complexes [53]. The key to *in situ* polymerization is appropriate dispersion of the filler in the monomer. This often requires organic modification of the particle surface or the metal precursors to improve their wettability with the monomer.

### 2.3. Sol-Gel Method

This is the typical bottom-up method combined with *in situ* formation of the nanofillers and *in situ* polymerization by using a sol-gel technique. A number of attempts to synthesize various types of filler/polymer nanocomposites, wherein inorganic filler phases with a dimension in the range from several angstroms to several nanometers are homogeneously distributed, *i.e.* organic/inorganic molecular hybrid materials, have been carried out by this method employing reactions of metal alkoxides [2,3,4,5,6,7,8,9,10,12,13,14,15,16,17,54,55,56,57,58,59]:

Si(OR)_4_ + 4 H_2_O → [Si(OH)*_x_*(OR)_4-*x*_ + *x*ROH] → SiO_2_ + 4 ROH + 2 H_2_O (1)

Here, Equation (1) expresses the hydrolysis and polycondensation reactions of tetraalkoxysilane as one of the typical sol-gel reactions used for synthesizing silica/polymer molecular hybrid materials. With the recent development of this sol-gel technology, nanocomposites and molecular hybrid materials have been extensively studied by many research groups. By using this technology, it is possible to disperse inorganic fillers with a dimension shorter than the molecular chain length of the matrix polymer. However the polymer species used as an organic domain of the hybrid materials in this sol-gel technique is limited to polymers possessing hydrogen bond acceptor groups that can form hydrogen bonds with the hydroxyl groups on the inorganic filler surface, such as alcohol- and water-soluble polymers. Furthermore, the conditions of the sol-gel reactions employed have a dramatic effect on the structure of the inorganic network formed, which makes it difficult to control the size and arrangement of the inorganic domain in the hybrid materials at the molecular level. The rigid limitation in the selection of polymers and the severe difficulty in synthesizing hybrid materials with stable quality and high reliability are a bottleneck in the stable supply and industrial application development of hybrid material products.

Recently, as a novel sol-gel technology to solve the above-mentioned bottleneck, a synthesis method of hybrid materials of a segmented-polymer and silica (Site Selective Molecular Hybrid Method) has been developed by using silane-modified polymers wherein oligomers of alkoxysilane were introduced selectively into appropriate sites in each polymer [58,59]. This method allows the use of polymer systems without any interactions with metal alkoxides, which were previously not suitable for the conventional sol-gel technology. The greatest advantage of this method is that it is possible to control the microstructure in the hybrid material according to its target application. For example, this technology has been applied to the improvement of the properties of polyurethane [58,59]. In general, the rubber elastic properties of segmented urethane block copolymers are caused by its two-phase microstructure in which hard-segments separate from the soft-segments to form domains. By the site selective molecular hybrid method, a hybrid material possessing dispersed hybrid domains of the hard-segment interpenetrating with silica in the soft-segment continuous phase could be synthesized. It has been reported that this hybrid material exhibited good mechanical and thermal properties because the hybrid domains are much harder and more heat resistant than the original hard-segment, while maintaining the flexibility of the soft-segment [58,59].

For the above-mentioned bottom-up approaches, many complex reactions have to be performed carefully using various kinds of chemical agents in the synthesis processes. Therefore, elaborate facilities and process control for the polymerization reactions and the disposal of chemical wastes discharged from the process are essential for the practical operation of these processes on an industrial scale.

### 2.4. Direct Mixing of Polymer and Nanofillers

Direct mixing of a matrix polymer and nanofillers is a top-down approach based on the breakdown of aggregated fillers during the mixing process. This type of method is suitable for fabricating polymer-based composites containing nano- or sub-micron-sized fillers with a dimension one or two orders of magnitude larger than that of the filler domains dispersed in the molecular hybrid materials. There are two general ways of mixing the polymer and fillers. The first is mixing a polymer, in the absence of any solvents, with nanofillers above the softening point of the polymer (melt-compounding method) [18,19,20,21,22,23,24,25]. The second is mixing the polymer and fillers as in a solution (solution-mixing method) [24,25,35,36,67,68,69,70].

**Figure 1:**
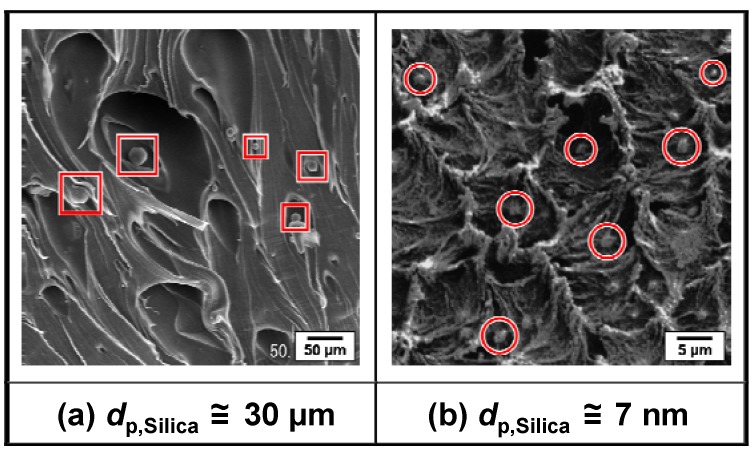
SEM micrographs of selected areas of the silica/PFA composites fabricated directly by melt-compounding PFA with 2.8 vol % unmodified silica additives with different sizes. (a) Fused silica particles with mean diameter of around 30 μm or (b) fumed silica powders with mean primary diameter of 7 nm were used as the additive for fabrication of each composite. The bright spots enclosed by squares are examples of isolated large silica particles distributed in the PFA matrix. The bright regions enclosed by circles are examples of micron-sized clusters of silica nanopowders. Adapted from Tanahashi, 2009 [64] and Tanahashi *et al.*, 2009 [75].

The melt-compounding method takes advantage of well established polymer processing techniques. In this kind of method, the shear stress (hydrodynamic force) induced in the polymer melt by melt-compounding is employed for the breakdown of aggregated fillers to the nano-scale. In general, the dispersion of inorganic fillers in the matrix polymer depends largely on the internal shear stresses induced by viscous drag on the fillers during melt-compounding. Previously, for example, some research groups have taken the dispersion of carbon black agglomerates and have proposed two mechanisms for describing the dispersion of the agglomerates [71,72]. In the rupture model [71], rupture occurs along a cross section in the agglomerate wherein the number of contact points of each primary particle with its neighbors is very low. The agglomerate cleaves into two nearly equal parts. In the “onion peeling” model [72], the stresses generated at the agglomerate surface are large enough at any point on the surface to remove a primary particle or a group of primary particles (an aggregate) from the surface of the larger agglomerate. The removed aggregates form a cloud around the initial agglomerate, partially shielding it from further size reduction. Reduction in agglomerate size occurs as aggregates are swept from the cloud and fresh aggregates from the agglomerate replace them. In either dispersion model, when the shear stress is larger than some critical threshold value for breaking down agglomerates of fillers, a dispersive action will occur inside the kneaded polymer melt [21,73,74]. Typical examples of the dispersion states of silica additives with different particle sizes in a completely hydrophobic perfluoropolymer, poly(tetrafluoroethylene-*co*-perfluoropropylvinylether) (PFA), are shown in Figure 1 (a) and (b). Figure 1 (a) shows a scanning electron microscope (SEM) micrograph of the silica/PFA composite fabricated through the direct melt-compounding of PFA with micron-sized fused silica (mean particle size: around 30 μm) without surface modification [64]. Figure 1 (b) shows the SEM micrograph of the composite fabricated by melt-compounding the PFA with unmodified nano-silica (fumed silica with mean primary diameter, *d*_p,Silica_, of around 7 nm) in powder form [64,75]. The volume fraction of silica, *V*_f,Silica_, in these two composite samples was held fixed at 2.8%. In the case when micron-sized silica was used as the additive in the PFA-matrix composite, the distribution of isolated silica particles in the matrix phase was observed. On the other hand, in the case when nano-sized silica was used as the additive, the dense agglomerates of silica nanopowders could not be broken down and they remained as micron-sized particle-clusters in the PFA matrix.

**Figure 2:**
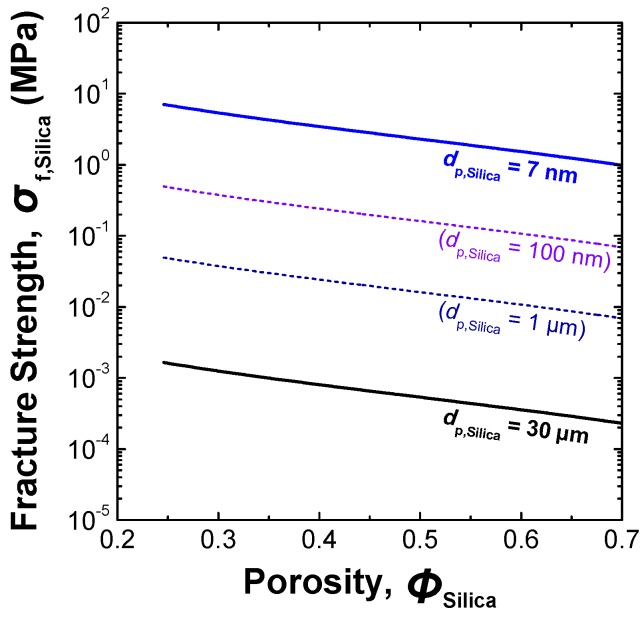
Relationship between the fracture strength of silica agglomerates and their porosity.

A comparison of Figure 1 (a) and Figure 1 (b) shows that the agglomerate strength of fillers exerts an influence on the dispersion state of fillers in the polymer matrix fabricated by the direct melt-compounding method. Figure 2 shows the agglomerate strengths of spherical silica particles with different sizes estimated as a function of the porosity of the agglomerate, using the following Rumpf’s equation [76]:

(2) $$\sigma_{\text{f}}=\frac{9}{8}\left(\frac{1-\phi }{\phi }\right)\frac{A_{\text{H}}}{24\cdot a^{2}\cdot d_{\text{p}}}$$

Here, *σ*_f_ is the fracture strength of the packing structure of a spherical particles agglomerate, *φ*; is the porosity of the packing structure, *a* is the distance between the surfaces of the two particles in the agglomerate, and *d*_p_ and *A*_H_ are the primary diameter and the Hamaker constant of the particles respectively. Equation (2) was derived as the theoretical tensile strength of a packed agglomerate of spherical particles on the basis of the binding force acting between two particles forming the agglomerate. For the estimation of *σ*_f_ for a silica agglomerate, the mean sizes of the silica primary particles used as the additive for the fabrication of the PFA matrix composites (*d*_p,silica_), 7 nm and 30 μm, were substituted for *d*_p_ in Equation (1). The value of *A*_H_ for fused silica in vacuum or air has been measured and calculated by several research groups [77,78,79]. There is a slight scatter, but most of the reported values are on the order of 10^−20^ Joules. Thus, their average, 5.5 × 10^−20^ J, was used as an approximate value of *A*_H,Silica_ for silica particles forming the agglomerate. For the value of *a*, the adhesion separation distance, which is often taken to be around 0.4 nm (single-ångström-ordered constant) for particles in intimate but chemically unbonded contact with a surface [79,80,81,82,83], was adopted. In Figure 2, the estimated agglomerate strengths for *d*_p,silica_ = 100 nm and 1 μm are also shown as comparison data.

**Figure 3:**
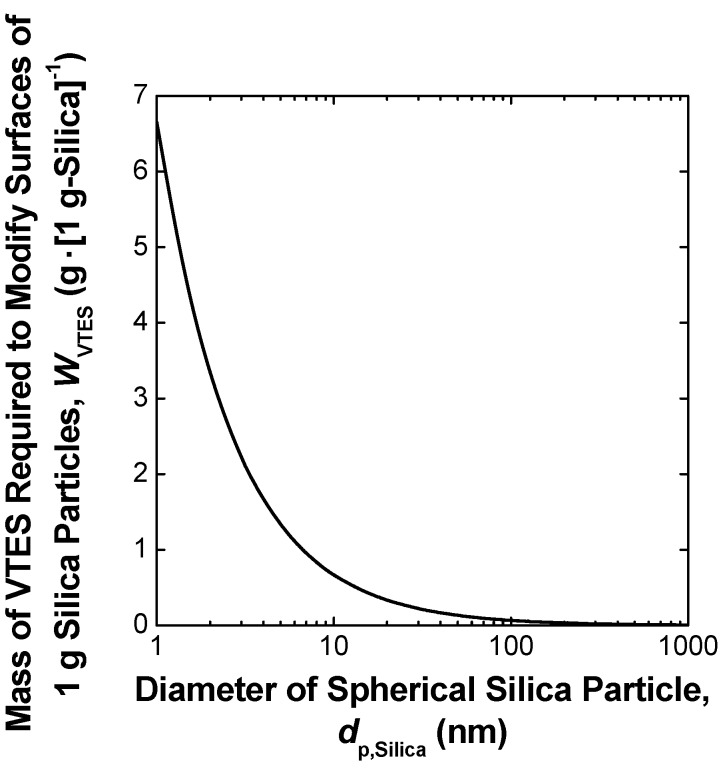
Mass of VTES required to modify surfaces of 1 g spherical silica nanoparticles.

It is expected from Figure 2 that the strength of the agglomerate of nano-sized silica particles is much weaker than that of the agglomerate of micron-sized silica particles. As shown in Figure 1 and Figure 2, when micron-sized silica was used as the additive, the hydrodynamic force induced in the kneaded PFA melt exceeds the cohesive force acting between the silica particles forming the agglomerate, resulting in the dispersion of the isolated large silica particles in the PFA matrix. However, the cohesive force between silica nanoparticles is so large that it is difficult to disperse the nanoparticles in the PFA using the direct melt-compounding method. This is a limitation of the simple melt-compounding method. Therefore, chemical modification of inorganic nanofillers from having a hydrophilic surface to a lipophilic one is used to achieve uniform dispersion of nanofillers that have hydroxyl-rich surfaces, such as silica, in hydrophobic polymers [26,27,28,30,31,33,34]. However, the surface area of the dispersed nanofillers is so large that a surface modifier, such as a silane coupling agent, is required for nano-dispersion on an industrial scale. Figure 3 shows an example of the relationship between the required mass of the surface modifier for uniform dispersion of inorganic particles and the particle size. The total amount of the monomolecular layer of vinyltriethoxysilane (VTES), a typical silane coupling agent, grafted onto 1 g of spherical silica nanoparticles (*W*_VTES_) was calculated from the specific gravities of silica (2.2 [84]) and VTES (0.9 at 298 K [26]), molecular weight of VTES (190.3 [26]), and its minimum covering area on the silica surface (410 m^2^·g^−1^ [26]). In the case when the diameter of the silica particles, *d*_p,Silica_, becomes smaller than 100 nm, the value of *W*_VTES_ rises sharply with decreasing particle size. In particular, the mass of VTES required to modify the surface of silica particles with *d*_p,Silica_ less than 6 nm exceeds that of the silica particles themselves.

In addition, it is extremely difficult to fabricate a silica/perfluoropolymer nanocomposite, even using the surface modification approach, because of the low chemical affinity (poor wettability) between the completely hydrophobic perfluoropolymer molecules and the silica nanoparticles that have hydrophilic surfaces. Recently, a melt-compounding method that combines shear mixing with ultrasonic mixing has been proposed as a unique top-down approach without any surface modification of the nanofillers [85]. It has been found that aggregated nanoparticles of titanium oxide could be highly dispersed into some kneaded polymer melts in extremely short times, less than one second, by utilizing a dynamic pressure field formed *via* ultrasonic waves. However, because ultrasonic waves are attenuated considerably in viscous polymer melts, it would be difficult to achieve uniform dispersion of the nanofillers into the polymer melts on a large scale.

When the solution-mixing method is concerned, the nanofillers are predispersed in a solution of the polymer, followed by evaporation of the solvent from the filler/polymer solution, which sometimes allows the fillers to be finely dispersed in the polymer matrix [24,25,35,36,67,68,69,70]. Because the shear force induced in the filler/polymer solution during the mixing process is much lower than that induced in the kneaded polymer melt in the absence of solvents, a predispersion of nanofillers in the solution is often achieved with the aid of an external force such as ultrasonic waves [24,25,35,67,68,69,70], as well as a surface modification of the fillers [35,36]. This method allows surface modification on the nanofillers without drying. In addition, an ultrasonic wave can be easily loaded to a filler/polymer solution with much lower viscosity than a polymer melt in the absence of any solvents. In terms of these issues, the solution-mixing method has an advantage over the melt-compounding method. However, in this method, polymers that are insoluble in conventional low boiling point solvents, such as polyethylene, cannot be used as the matrix for composites [32]. The limitation of the polymer matrix is one of the disadvantages in the solution-mixing method.

## 3. Simple Melt-Compounding without Surface Modification of Nanofillers Proposed by the Author’s Research Group

The author and coworkers have proposed an advanced version of the melt-compounding approach without requiring any surface modification of nano-sized inorganic particulate fillers, as a simple and versatile method for the fabrication of filler/polymer nanocomposites with a wide composition range. The proposed method has great advantages over either the intercalation method, the *in situ* polymerization method, the sol-gel method or the solution-mixing method. First, this method is environmentally benign due to the absence of organic solvents, organic surfactants, and a variety of specific chemical substances required for the complicated polymerization and sol-gel reactions. Second, it is compatible with current industrial processes, such as extrusion and injection molding, and suitable for large-scale production of the nanocomposite products. Third, it allows the use of polymers that were previously not suitable for *in situ* polymerization, sol-gel reaction or solution-mixing.

### 3.1. Concept

**Figure 4:**
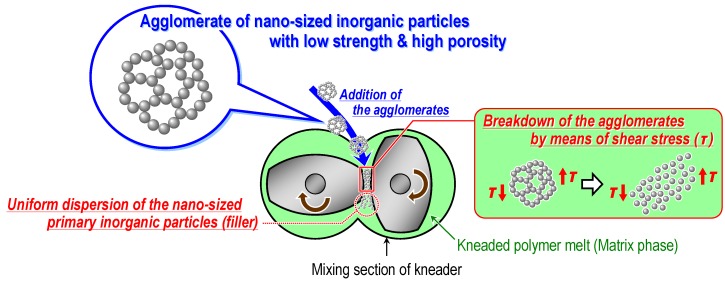
Conceptual illustration of the fabrication of filler/polymer nanocomposites by the direct melt-compounding of the polymer with prepared strength-controlled agglomerates of inorganic nanoparticles with high porosity.

As mentioned in section 2.4, not only the shear stress induced in the kneaded polymer melt but also the critical threshold value necessary for breaking down the agglomerates of nanofillers are the main factors that control the dispersion state of the fillers in the melt-compounding method. Therefore, the author’s research group has focused on the fracture strength of agglomerates of the dispersed nanoparticles (required stress for the breakdown of the agglomerates into nano-sized primary particles) [60,61,62,63,64,65,66]. For this method, the preparation stage of the strength-controlled agglomerates with a porous structure (the first stage) was combined with the melt-compounding process (the second stage). Reducing the fracture strength of the agglomerate of nanoparticles before the melt-compounding stage is one of the key strategies for obtaining a uniform nano-dispersion of the particles using this method. If the agglomerate strength is sufficiently low, it is possible to break down the agglomerates and to achieve a uniform dispersion of the nanoparticles without surface modification in various polymer compositions by the shear stress induced during the melt-compounding process, as illustrated in Figure 4.

As shown in Equation (2) and Figure 2, the fracture strength of an agglomerate of nanoparticles, σf, decreases with increasing agglomerate porosity, *φ*. The relationship between *σ*_f_ and *φ* indicates that the fracture strength of a silica agglomerate can be reduced by altering the particle arrangement in the agglomerate from a close-packed structure (dense agglomerate) to an open-packed one (loose agglomerate).

In the authors’ studies [60,61,62,63,64], nano-sized spherical particles of silica were selected as the dispersed filler for the fabrication of various filler/polymer nanocomposites. A commercially available aqueous colloidal solution of spherical silica with *d*_p,Silica_ of 190 nm was used as the starting material for the preparation of an open packing agglomerate of nano-sized silica particles with low strength and high porosity. On the basis of the chemistry of colloidal silica [84] and the DLVO (Derjaguin-Landau-Verwey-Overbeek) theory on the stability of a colloidal dispersion system [86,87], the packing structure of the agglomerate was controlled, ensuring open packing with a large amount of porosity. In the following section, the experimental techniques and the main findings of the previous studies carried out by the author and coworkers [60,61,62,63,64] are reviewed together with the results on the dispersion state of silica particles into various polymers by melt-compounding each polymer with the prepared agglomerates.

### 3.2. Experimental Techniques and Main Findings of Authors’ Previous Studies

#### 3.2.1. Preparation of loose silica agglomerate in the first stage

**Figure 5:**
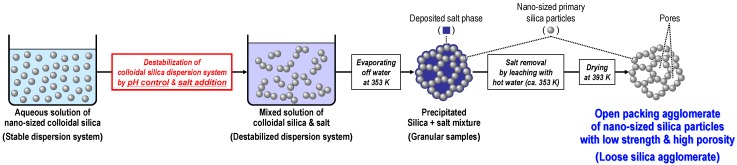
Schematic illustration of the preparation method of an open packing agglomerate of nano-sized silica particles with low strength and high porosity.

The preparation method for loose silica agglomerates is shown in Figure 5. In a colloidal silica solution destabilized by pH control and the addition of salts, the colloidal silica particles coagulate, resulting in the formation of loose silica agglomerates with a large number of pores. Evaporating off the water yielded lumps of precipitated agglomerates of silica nanoparticles mixed with deposited salt (silica + salt mixtures). The resultant granular samples of the silica + salt mixture were immersed in hot water (around 353 K) to leach the salt phase. Finally, highly porous silica agglomerates were obtained by drying the sample at 393 K. In the authors’ study [60,61,62,63,64], a commercially available aqueous colloidal solution of around 40 wt% spherical SiO_2_ with *d*_p,Silica_ = 190 nm (MP-2040, Nissan Chemical Industries, Ltd., pH 9.3), potassium bromide (KBr) and nitric acid, were used as the starting material, salt and pH controller for the preparation of the silica agglomerates, respectively.

**Figure 6:**
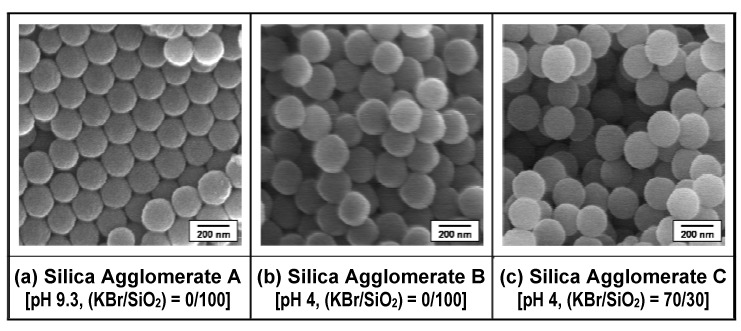
SEM micrographs of selected areas of the surfaces of silica agglomerates A, B, and C prepared from three types of colloidal silica solutions (a) without pH control or KBr addition (pH 9.3, (KBr/SiO_2_) = 0/100), (b) with pH control alone (pH 4, (KBr/SiO_2_) = 0/100), and (c) with pH control and KBr addition (pH 4, (KBr/SiO_2_) = 70/30), respectively. (KBr/SiO_2_) indicates the ratio of vol.% of KBr to that of SiO_2_ in the colloidal solution. Adapted from Tanahashi *et al.*, 2007 [62].

Figure 6 shows SEM micrographs of selected areas on the surfaces of the silica agglomerates prepared under three different conditions of the colloidal silica solution. An orderly arranged structure (regular close packing) was observed in silica agglomerate A prepared from the colloidal solution without pH control or KBr addition, as shown in Figure 6 (a). Figure 6 (b) and Figure 6 (c) show the packing arrangements in the silica agglomerates B and C prepared from the pH-controlled colloidal silica solutions (pH 4) without and with KBr addition, respectively. The steric arrangement of the nano-sized primary silica particles in the silica agglomerate could be varied from an ordered structure (regular close packing) to a disordered structure (random loose packing) by controlling the pH and salt concentration in the colloidal solution. In particular, the packing structure of the silica agglomerate prepared from the colloidal solution with pH control and KBr addition appears to be the most disordered of the three agglomerates. The pore structure and fracture strength of agglomerates A, B and C, characterized using mercury intrusion porosimetry and microcompression tests, respectively, are summarized in Table 1. The porosity (*φ*_Silica_), the mean value of the pore diameter (*D*_P,Silica_) and the standard deviation of the pore diameter (*σ*_P,Silica_) are shown in this table as the former characteristic, and the mean fracture strength (*σ*_f,Silica_) is shown as the latter one. In addition, the porosity and strength of a commercially available fine porous silica gel are also listed to compare with those of the prepared silica agglomerates A to C. The strength of the silica agglomerates prepared from the colloidal silica solution is lower than that of the commercially available fine porous silica gel even though the porosities of all the silica agglomerate samples are lower than that of the porous silica gel. The pore size and the pore distribution in the prepared agglomerate become larger and broader in the order of the agglomerates A, B, and C. Under the solution conditions of pH 4 and (KBr/SiO_2_) = 70/30, high porosity with limited contact between the primary silica particles in agglomerate C could be achieved, resulting in an extreme reduction in the fracture strength. By controlling the conditions of the colloidal silica solution (the starting material) *via* pH control and KBr addition, the packing arrangement of the primary silica particles in the prepared agglomerates could be varied from an ordered structure (regular close packing) with relatively higher strength to a disordered structure (random loose packing) with relatively lower strength. These results imply that the destabilizing step of the colloidal silica solution controls the pore structure and strength of the prepared silica agglomerates.

It was clarified that destabilization of the colloidal silica solutions in the first stage is one of the key strategies for preparing loose silica agglomerates with low strength. On the basis of colloidal stability, the formation of the packing structure of the silica agglomerates in the colloidal silica solution can be explained as follows. The chemistry of colloidal silica [84] suggests that the combined approach of pH control and KBr addition leads to collisions and hence bridging between the silica nanoparticles at a limited number of surface sites, which is essential for the fabrication of open packed silica agglomerates.

**Table 1:** Characteristics of pore structure and strength of prepared silica agglomerates A to C and a certain commercially available product of fine porous silica gel. Data from Tanahashi *et al.*, 2006 [60], Watanabe *et al.*, 2006 [61] and Tanahashi *et al.*, 2007 [62].

Sample	Pore structure			Mean fracture strength, *σ*_f,Silica_ (MPa)
	Mean pore diameter, *D*_P,Silica_ (nm)	Standard deviation of pore diameter, *σ*_P,Silica_ (nm)	Porosity, *φ*_Silica_	
Silica agglomerate A	40.15	14.94	0.34	3.3
Silica agglomerate B	51.89	19.82	0.39	0.8
Silica agglomerate C	60.94	26.27	0.45	0.5
Commercially available product of fine porous silica gel	Unknown	Unknown	0.75	10.0

First, collisions between the silica nanoparticles in the colloidal solution caused by pH control and salt addition were examined on the basis of the interaction between the silica nanoparticles in the colloidal solution. According to the DLVO theory [86,87], the main forces acting on the colloidal particles in a liquid medium are the attractive van der Waals (VDW) interaction and the repulsive electrostatic double-layer (EDL) interaction forces. Thus, the total interaction energy between a pair of approaching particles, *E*_T_, is given by the sum of these two forces: *E*_A_ + *E*_R_, where *E*_A_ is the energy of the VDW interaction, and *E*_R_ is the energy associated with the repulsive EDL interaction. The interaction energies of *E*_A_ [88] and *E*_R_ [89] acting between two spherical particles can be expressed as:

*E*_A_ = −(*A*_H_/6)·[*d*_p_^2^/2*H*(*H* + 2*d*_p_) + *d*_p_^2^/2(*H* + *d*_p_)^2^ + ln {1 − *d*_p_^2^/(*H* + *d*_p_)^2^}] (3)

*E*_R_ = *π*·*ε*·*d*_p_·*ψ*_0_^2^·ln [1 + exp (−*κ*·*H*)] (*d*_p_ >>*κ*^−1^) (4)

where *A*_H_ is the Hamaker constant (J), *d*_p_ the primary diameter (m) of the particles, *H* the distance between the surfaces of two particles (m), *ε* the permittivity of the liquid medium (F·m^−1^), *ψ*_0_ the electrostatic surface potential (V) and *κ* the Debye-Hückel parameter (m^−1^) (*κ*^−1^, the Debye length, is an index of the “thickness” or “extent” of the EDL). The parameters *ψ*_0_ (for an oxide particle, such as SiO_2_ and TiO_2_) [90] and *κ* [83,91] can be expressed as:

*ψ*_0_ = (2.303 *kT*/*e*)·(pH_0_ − pH) (5)

**Figure 7:**
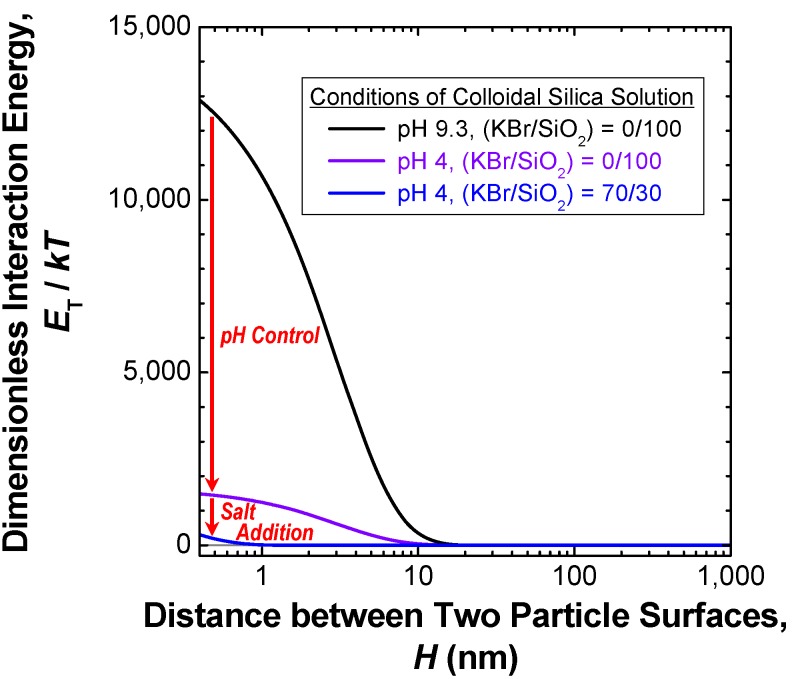
Effects of salt addition and pH control on the dimensionless energy *versus* distance profile of the DLVO interaction for the colloidal silica solution with a mean primary diameter of 190 nm at the beginning stage of the evaporation step of the solution at 353 K. Data from Tanahashi *et al.*, 2007 [62].

*κ* = (2*e*^2^*n*_0_*z*^2^/*εkT*)^1/2^ (6)

where *k* is the Boltzmann’s constant (1.38066 × 10^−23^ J·K^−1^), *T* the absolute temperature (K), *e* the electronic charge (1.6021773 × 10^−19^ C), pH_0_ the isoelectric point of the particle and *n*_0_ the bulk concentration (number density) of ions with valence *z* in the liquid medium (m^−3^). For highly charged surfaces in a dilute electrolyte (*i.e.*, a long Debye length), there is a strong long-range repulsion that peaks at some distance, usually between 1 and 4 nm [79]. This maximum value of *E*_T_ is referred to as the “energy barrier”. It is thought that the dispersion/coagulation behavior of colloidal particles is controlled by the balance between the energy barrier between the particles and the kinetic energy of the particles. If the energy barrier is much larger than the kinetic energy of individual particles, it may be too high for the particles to surmount, which leads to a stable dispersion state. Conversely, when the energy barrier becomes too low, the colloidal particles collide in a liquid medium; the colloid is then referred to as being “unstable.” Under the assumption that the dispersion/coagulation behavior of the colloidal particles during evaporation of the solvent (water) at 353 K immediately after KBr addition and pH control determines the packing structure of the silica agglomerates, the values of *E*_T_ at the beginning of the evaporation step were calculated by substituting the physicochemical properties of the colloidal silica solution containing the prescribed amount of KBr (*i.e.*, pH, pH_0_, *A*_H_, *ε*, *n*_0_ and *z* = 1) into Equations (3)–(6). The relationships between the calculated interaction energy acting between two silica particles both with a *d*_p,Silica_ of 190 nm in the colloidal solution at 353 K divided by the kinetic energy of the particle at this temperature (*E*_T_/*kT*) and the distance between the surfaces of two particles (*H*) are shown in Figure 7 as a function of pH and salt (KBr) concentration in the solution [62].

It is seen from Figure 7 that the commercially available solution of colloidal silica at pH 9.3 without KBr addition is in a stable dispersion state. Thus, the aqueous solvent of the colloidal solution is evaporated in a good dispersion state, resulting in the formation of the close packing structure (the ordered arrangement) shown in Figure 6 (a), wherein the Gibbs free energy of the colloidal system is minimized. This phenomenon has been applied to micropatterning of spherical particle assemblies [92,93,94,95]. The exact opposite approach, capable of destabilizing the colloidal system, can be employed for forming a disordered arrangement of silica nanoparticles. As shown in Figure 7, the energy barrier is significantly lowered by pH control and KBr addition, resulting in random collision of the particles.

**Figure 8:**
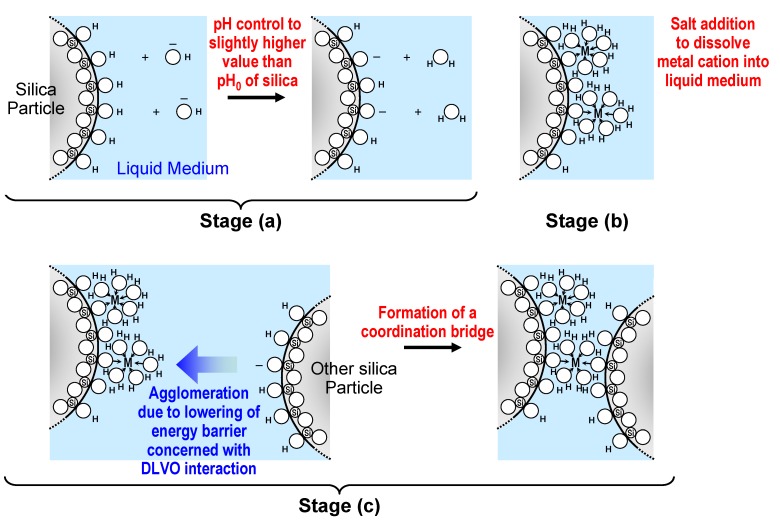
Schematic illustration of the presumed bridging process between silica particles through coordination with flocculating metal cations with a coordination number of 6. Open circles represent oxygen atoms. Adapted from Tanahashi *et al.*, 2007 [62].

Next, the effects of pH control and salt addition on the formation of the open packing structure were examined from the viewpoint of the “bridging factor” in the preparation of loose silica agglomerates. According to the chemistry of colloidal silica [84], the random collision of silica fine particles in an aqueous colloidal solution destabilized by pH control and salt addition may lead to bridging between the particles, as shown in Figure 8 [62]. This figure shows schematically the bridging process between silica particles through coordination with flocculating metal cations with a coordination number of 6. When the pH of the aqueous colloidal silica solution is set to 4, which is slightly higher than the pH_0_ of silica, protons are dissociated from some of the silanol groups on the silica particle surface, resulting in the formation of negative surface sites (Stage (a) in Figure 8). The number of negative charges (the negative surface sites) per unit area of the silica surface is reduced with decreasing pH within the pH range from the pH_0_ of silica to 7, as indicated by the following ionization reaction of surface silanol groups:

≡Si−OH → ≡Si−O^−^ + H^+^ (7)

When salts, such as KBr, are added to the solution, the metal cations that dissociate from these salts are surrounded by oxygen atoms with six water molecules of hydration. Such hydrated potassium ions are adsorbed at the negative surface sites on the silica particle forming a neutral complex (Stage (b) in Figure 8). Because the colloidal silica solution becomes destabilized by pH control and salt addition, the dispersed silica particles in the solution begin to collide randomly, as mentioned above. The collision with a second particle permits metal cations to coordinate with the oxygen atoms of silanol and surface-bonded water, forming a coordination bridge between the particles (Stage (c) in Figure 8).

In the present study, this process leads to bridging *via* potassium ions at a limited number of surface sites on the silica nanoparticles in the destabilized colloidal silica solution, resulting in the formation of three-dimensional networks of silica particles with high porosity, as shown in Figure 6 (c).

The results of the packing structure and the fracture strength of the prepared silica agglomerates shown in Figure 6 and Table 1 show that the combination of a reduction in the pH of the colloidal silica solution and the presence of metal cations in the solution serves to control the limited bridging between the silica particles in the agglomerate and allows the agglomerate strength to be greatly reduced. This approach to colloidal solutions *via* pH control and salt addition may enable control of the pore structure and fracture strength of the agglomerates of various types of metal oxide nanoparticles. The author’s research group has made some attempts to prepare titania and alumina agglomerates with a pore structure by controlling the stability of their dispersion systems of spherical colloidal nanoparticles [96]. In view of the extension of application range of the proposed method for fabrication of the filler/polymer nanocomposite systems illustrated in Figure 4, it is significant to verify the applicability of this preparation method for loose agglomerate to non-spherical filler systems, such as montmorillonite and mica, as well as the above-mentioned inorganic spherical nanoparticles. In the future, it is expected to demonstrate the universality of the preparation method for loose agglomerate experimentally.

#### 3.2.2. Melt-compounding of polymer matrices with silica agglomerates in the second stage

**Figure 9:**
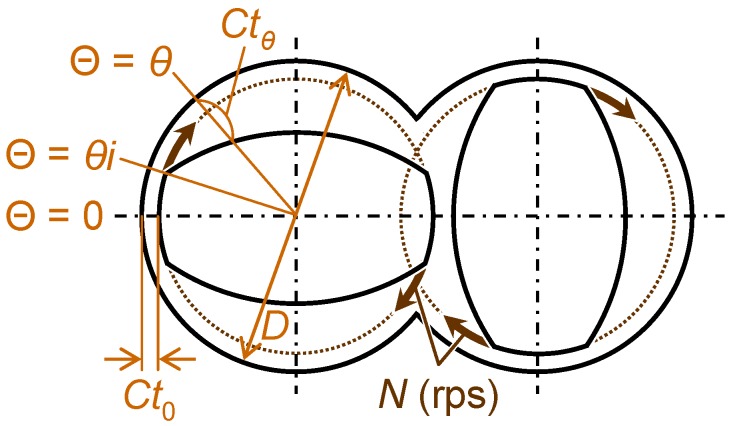
Schematic illustration of a cross section of the mixing section in the twin-screw intensive batch kneader used in the authors’ previous studies. Adapted from Tanahashi *et al.*, 2006 [60] and 2007 [62].

In addition to the control of the packing structure and fracture strength of the silica agglomerate in the first stage, the shear stress acting on the agglomerates during the melt-compounding of polymer matrices (*τ*_Polymer_) is also an important factor for uniform dispersion of primary silica particles into each matrix. If the shear rate is given as the melt-compounding condition of a matrix polymer and the shape of the screw in the kneader used for the melt-compounding are given, the value of *τ*_Polymer_ can be calculated from the melt viscosity of the polymer. In the authors’ previous studies [60,61,62,64], a intensive batch kneader (Labo Plastomill, KF70 model, Toyo Seiki Seisaku-sho, Ltd.), illustrated in Figure 9, was used for melt-compounding of each of the following four polymers: Poly(ethylene-*ran*-vinylalcohol) (EVOH), polycarbonate (PC), polystyrene (PS) and poly(tetrafluoroethylene-*co*-perfluoropropylvinylether) (PFA) with loose silica agglomerates prepared under the conditions summarized in Table 2. According to Takase *et al.* [97], when a mixer with an ellipsoidal shaped twin-screw, as illustrated in Figure 9, is used for melt-compounding, the maximum and minimum shear rates, ${\dot{\gamma }}_{\max}$ and ${\dot{\gamma }}_{\min}$, can be, respectively, derived as:

(8) $${\dot{\gamma }}_{\max}\;=\;\pi (D-2Ct_{0})N/Ct_{0}\;(0\leq \Theta \leq \theta i)$$

(9) $${\dot{\gamma }}_{\min}\;=\;\pi (D-2Ct_{\theta })N/Ct_{\theta }\;(\Theta =\pi /2)$$

by using the screw rotational speed, *N* (rps), the internal diameter of the cylinder, *D* (m) and the clearances between the inner wall of the cylinder and the rotor surface at angles Θ of 0 and *θ* in the mixing section of the kneader, *Ct*_0_ (m) and *Ct_θ_* (m), respectively.

**Table 2:** Experimental conditions of melt-compounding of various polymers with loose silica agglomerates in the authors’ previous studies. Data from Tanahashi *et al.*, 2006 [60], Watanabe *et al.*, 2006 [61], Tanahashi *et al.*, 2007 [62] and Tanahashi, 2009 [64].

Matrix polymer	Volume fraction of silica, *V*_f,Silica_ (%)	Melt temp. (K)**	High rotational speed mode		Low rotational speed mode		Number of cycle***
			Rotational speed (rps)	Time (s)	Rotational speed (rps)	Time (s)	
EVOH	2.7	463−496	3.3	20	0.3	180	4
PC	1.0*	513−543	3.0	20	0.3	180	4
PS	2.5, 4.9	453−478	3.3	20	0.3	180	4
PS	9.8	453−483	3.3	20	0.3	180	4
PFA	2.8, 14.4	613−643	4.0	20	0.3	240	5
PFA	27.1	618−643	3.3	20	0.3	180	4

* The loose silica agglomerate prepared from colloidal silica solution with pH control and KBr addition (pH 2 and (KBr/SiO_2_) = 70/30) was used as the filler only in the case of the PC matrix. In the cases of the other polymer matrices, the loose silica agglomerate C (see Figure 6 (c) and Table 1) prepared from colloidal silica solution with pH control and KBr addition (pH 4 and (KBr/SiO_2_) = 70/30) was mixed with each polymer melt.

** Temperature of the kneaded polymer melts was changed within each range mentioned in this column during the melt-compounding processes.

*** The melt-compounding processes were operated under the shear conditions where the screw rotational speed was changed cyclically between a wide range from the high mode to the low one.

**Figure 10:**
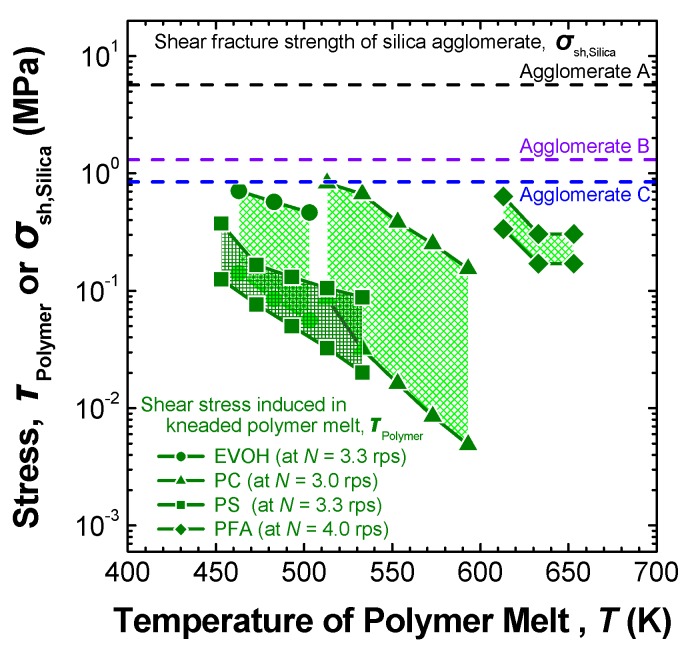
Comparison of shear stresses induced in various polymer melts during melt-compounding at each screw rotation speed with the shear fracture strength of the silica agglomerates A, B and C. Adapted from Tanahashi *et al.*, 2006 [60], Watanabe *et al.*, 2006 [61] and Tanahashi *et al.*, 2007 [62].

The maximum and the minimum values of *τ*_Polymer_ induced in each of EVOH, PC, PS and PFA melts were calculated as a function of the melt temperature and the screw rotational speed. As some examples of the calculated results for *τ*_Polymer_, the ranges of *τ*_Polymer_ for these four polymer melts are plotted in Figure 10 against the melt temperature under each appropriate condition of high screw rotational speed (*N* = 3–4 rps, see Table 2) that resulted in good dispersion of silica [60,61,62]. The pair of upper and lower lines corresponds to the distribution of *τ*_Polymer_ from the minimum value to the maximum one for each polymer melt.

Equations (8) and (9) indicate that the shear stress induced in general thermoplastic polymer melts increases with increasing screw rotation speed. However, simultaneously, the shear stress decreased as a result of the internal heat generation at high rotor rotation rates and the concomitant increase in the melt temperature, as shown in Figure 10. Thus, it is necessary to consider the screw rotation speed as well as the melt viscosity in selecting the conditions for melt-compounding. In the authors’ previous studies [60,61,62,64], to prevent the polymer melt temperature from rising excessively and to maintain the shear stress at a high level during melt-compounding, the screw rotational speed was set to alternate between a higher value for a relatively short-time period and then a lower value for a relatively long-time period, as shown in Table 2.

Figure 10 also plots the shear fracture strengths (*σ*_sh,Silica_) of the silica agglomerates A, B and C estimated from the results of *σ*_f,Silica_ listed in Table 1, using the following equation for estimating the shear strength [98,99]:

(10) $$\sigma_{sh,Silica}=\sqrt{3}\cdot \sigma_{f,Silica}$$

The shear fracture strength of the loose silica agglomerate C prepared from the colloidal silica solution with pH control and KBr addition (pH 4 and (KBr/SiO_2_) = 70/30) is one order of magnitude lower than those of the other two agglomerates A and B and is comparable to the maximum shear stresses induced in the EVOH, PC and PFA melts. If the loose agglomerates are blended with these three types of polymers in the mixer under these melt-compounding conditions, the agglomerates are sufficiently weak for them to be broken down in each polymer melt by the shear stress. This implies that a uniform dispersion of primary silica nanoparticles could be achieved in EVOH, PC and PFA by direct melt-compounding of each polymer with the prepared loose silica agglomerates. On the other hand, it is expected to be difficult to break down the silica agglomerates C in the PS matrix, as shown in Figure 10.

#### 3.2.3. Distribution of primary silica particles in various polymer matrices

**Figure 11:**
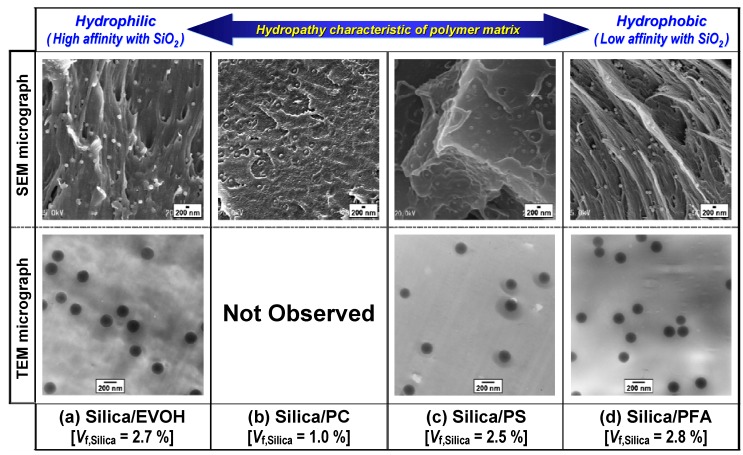
SEM and TEM micrographs showing dispersion states of silica nanoparticles in silica/polymer composites fabricated by melt-compounding each polymer with loose silica agglomerates prepared from the colloidal silica solution with pH control and KBr addition (pH 4 and (KBr/SiO_2_) = 70/30) or (pH 2 and (KBr/SiO_2_) = 70/30): (a) silica/EVOH composite (*V*_f,Silica_ = 2.7%) [60,61], (b) silica/PC composite (*V*_f,Silica_ = 1.0%) [61], (c) silica/PS composite (*V*_f,Silica_ = 2.5%) [60,61] and (d) silica/PFA composite (*V*_f,Silica_ = 2.8%) [61,62,64]. The bright spots in the SEM micrographs and the dark spots in the TEM micrographs are dispersed primary silica particles on the surfaces of the composites. Adapted from Tanahashi *et al.*, 2006 [60], Watanabe *et al.*, 2006 [61], Tanahashi *et al.*, 2007 [62] and Tanahashi, 2009 [64].

The silica dispersion state in each silica/polymer composite fabricated was characterized by SEM and transmission electron microscopic (TEM) observations. The SEM and TEM micrographs shown in Figure 11 show typical examples of the dispersion states of silica particles into (a) EVOH (*V*_f,Silica_ = 2.7%), (b) PC (*V*_f,Silica_ = 1.0%), (c) PS (*V*_f,Silica_ = 2.5%) and (d) PFA (*V*_f,Silica_ = 2.8%) matrices [60,61,62,64]. These composites were fabricated by melt-compounding each of the polymers having different hydropathy characteristics with appropriate amounts of the prepared highly porous silica agglomerate under the conditions tabulated in Table 2. Hydropathy characteristics of the polymer matrix are directly linked to the chemical affinity for the hydrophilic surface of the silica fillers, such as wettability at the polymer-silica interface. Poly(ethylene-*ran*-vinylalcohol) contains numerous hydrophilic vinylalcohol groups as one of its main components and has the highest affinity for silica out of the four hydrophobic polymers used in the author’s studies. The completely hydrophobic perfluoropolymer, PFA, has the lowest affinity with silica. Polycarbonate and PS are moderate affinity polymers. The former polymer has a larger polarity than the latter one. In the cases of the EVOH, PS, and PFA matrices, the loose silica agglomerate C prepared from colloidal silica solution with pH control and KBr addition (pH 4 and (KBr/SiO_2_) = 70/30) was used for the melt-compounding. Only in the cases of the fabrication of the silica/PC nanocomposite, a silica agglomerate with an open-packed structure (*D*_P,Silica_ = 60.01 nm, *σ*_P,Silica_ = 28.85 nm, *φ*_Silica_ = 0.44) that was prepared from a colloidal silica solution containing KBr ((KBr/SiO_2_) = 70/30) with pH 2 was used as the silica filler (see the footnote (*) on Table 2). The micrographs clearly show that the nano-sized primary silica particles are dispersed uniformly in all polymer matrices. Each nano-sized primary particle on the fracture surface of the silica/EVOH composite is isolated from the other primary particles. Similar images were obtained for the cases of PC and PS matrices. Although Figure 10 indicates that the silica agglomerate C in the kneaded PS melt cannot be broken down by the shear stress, as mentioned above, Figure 11 (c) shows that the uniform dispersion of the isolated primary silica particles was unexpectedly achieved in the PS. The exact reason for this is not known. In addition, Figure 11 (d) reveals that each primary silica particle with a hydrophilic surface is mono-dispersed and adheres to the fibrous fracture surface of the completely hydrophobic (water-repellent) perfluoropolymer PFA, despite the very low chemical affinity between the two materials. The author’s research group has also successfully obtained a silica/polypropylene (PP) nanocomposite in which uniform dispersion of silica nanoparticles with a diameter of 190 nm was achieved for *V*_f,Silica_ of 5% [63] and above by the same method. In general, when the inorganic fillers are dispersed into a polymer matrix by a conventional melt-compounding method, the dispersion state of the fillers is considered to depend strongly on the chemical characteristics of the polymer matrix, such as wettability with the filler surface [100]. Especially in the case when nonpolar polyolefins, such as PP, having strong hydrophobic properties, as well as PFA, is used as the matrix polymer, a good dispersion of nanofillers into these matrices is hard to achieve by simple melt-compounding [22,25,33,34]. For example, direct melt-compounding PP with several percent of silica nanoparticles resulted in high particle aggregation in the past [22,25,33]. However, it was clarified from Figure 11 [60,61,62,64] and the results on the silica/PP nanocomposites [63] that good dispersion of nano-silica can be achieved in a wide variety of polymer matrices, from a hydrophilic EVOH to an extremely hydrophobic polymer such as PFA and PP, regardless of the degree of wettability with the silica surface.

**Figure 12:**
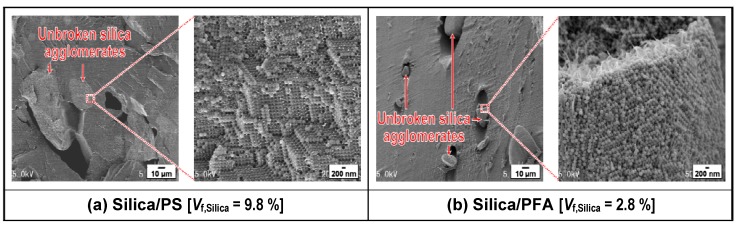
SEM micrographs showing dispersion states of silica nanoparticles in silica/polymer composites fabricated by melt-compounding PS or PFA with dense silica agglomerates A prepared from the colloidal silica solution without pH control or KBr addition: (a) silica/PS composite (*V*_f,Silica_ = 9.8%) and (b) silica/PFA composite (*V*_f,Silica_ = 2.8%). Adapted from Tanahashi *et al.*, 2007 [62].

**Figure 13:**
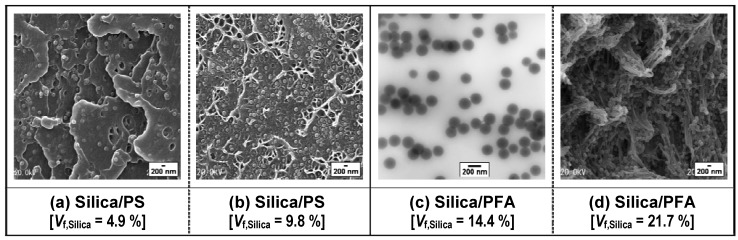
(a, b, d) SEM and (c) TEM micrographs showing dispersion states of silica nanoparticles in PS and PFA matrices for varying volume fractions of loose silica agglomerates C prepared from the colloidal silica solution with pH control and KBr addition (pH 4 and (KBr/SiO_2_) = 70/30): silica/PS composites (*V*_f,Silica_ = (a) 4.9 and (b) 9.8%) [60] and silica/PFA composites (*V*_f,Silica_ = (c) 14.4 and (d) 27.1%) [62]. The bright spots in the figures are dispersed primary silica particles on the surfaces of the composites. Adapted from Tanahashi *et al.*, 2006 [60] and 2007 [62].

Figure 12 shows SEM micrographs of selected areas of the fracture surface of the negative control composite sample fabricated by melt-compounding each of the PS and the PFA polymers with appropriate amounts of the close-packed silica agglomerates A with relatively high strength prepared from the colloidal silica solution without pH control or KBr addition. In both cases of PS and PFA matrices, the silica agglomerate A was so dense that the agglomerate could not be broken down and remained as micron-sized large particle-clusters in the kneaded polymer melt. Dispersion characteristics of silica particles in the same polymer matrix differ depending on the strength of the agglomerates used as the filler. For example, the dispersion states of the dense agglomerate A and the loose agglomerate C in the same PFA matrix can be compared by using the SEM images in Figure 11 (d) and Figure 12 (b). It is shown from these two figures that the dispersion state of the silica nanoparticles is much better in the case where the loose silica agglomerates were dispersed into the PFA by melt-compounding than in the case where dense silica agglomerates were used. These results support the relationship between the shear stress induced in the PFA melt and the shear strengths of the silica agglomerates A and C shown in Figure 10. The effect of the packing structure of the silica agglomerate on the dispersion state of silica in the PS matrix has a similar tendency to the case of the PFA matrix (see Figure 13 (b) and Figure 12 (a)). These results imply that pH control and KBr addition in the stage of silica agglomerate preparation from a colloidal solution have enabled the breakdown of the agglomerates by shear stress induced in the polymer melt during the melt-compounding and mono-dispersion of the primary silica particles into the polymer matrix.

In general, good dispersion of inorganic nanofillers in polymer matrices is especially hard to achieve by simple melt-compounding at a high filler content. It is significant to clarify the relationship between volume fraction of silica and its dispersion state in the silica/polymer composite fabricated by the authors’ method. The dispersion state of primary silica particles did not change with an increase in the silica volume fractions, *V*_f,Silica_, from 2.5 to 9.8% for the PS matrix [60] and from 2.8 to 27.1% for the PFA matrix [62], as shown in Figure 13 (a) and Figure 13 (b), respectively. The distance between two primary silica particles distributed separately in the PS and PFA matrices decreases with an increase in the silica volume fractions because the numbers of the particles in a unit volume increase with an increase in the volume fractions. Although conventional melt-compounding has been recognized to be unable to promote fine and homogeneous dispersion of the filler, particularly at a high filler content [32,33,34], up to 30 vol % silica nanoparticles without any surface modifications could be dispersed uniformly even in the completely hydrophobic PFA by the authors’ method [62].

## 4. Summary

In the authors’ studies, the dispersion of nano-sized silica particles with hydrophilic surfaces into various hydrophobic polymers was investigated by simple direct melt-compounding using loose silica agglomerates with low fracture strength. The packing structure and fracture strength of the agglomerates of silica nanoparticles could be controlled by destabilizing an aqueous solution of a colloidal silica dispersion system *via* pH control and salt addition. The preparation of the loose agglomerate of silica nanoparticles, which is a major key strategy for the proposed nanocomposite fabrication method, is expected to be applicable for the preparation of the agglomerates of other inorganic nanoparticles. Recently, an attempt has been made to produce loose silica agglomerates by the authors’ method on a pilot scale [65,66]. By the proposed method, silica particles with a mean diameter of 190 nm could be successfully dispersed in various polymer matrices, regardless of the degree of chemical affinity between the dispersed silica and the matrix polymer. This is one of the major advantages of the proposed method. Another advantage is that the method enables the uniform dispersion of nanoparticles into a polymer matrix, while maintaining their surface activity at a high level because the method involves no chemical reactions at the interfaces between the nanoparticle and the polymer phase. This implies that the prepared loose silica agglomerate is also a potential candidate for a catalyst support for nano-dispersion of the catalyst into the polymer matrix by direct melt-compounding. In view of these advantages, this approach is expected to become a simple and versatile method for fabricating filler/polymer nanocomposite materials with a wide volume fraction range of nanofiller and a variety of combinations of filler and polymer material species. Moreover, in the authors’ recent studies, it has been found that some of the silica/thermoplastic nanocomposites fabricated by the proposed method have some unique mechanical or thermal characteristics [63,64,75]. Therefore, the proposed method has not only a high productivity but also the potential capacity for fabricating high performance filler/thermoplastic nanocomposites with a wide composition range.
